# Baseline characteristics and event rates among anticoagulated patients with atrial fibrillation in practice and pivotal NOAC trials

**DOI:** 10.1016/j.dib.2017.08.010

**Published:** 2017-08-09

**Authors:** Peter A. Noseworthy, Xiaoxi Yao, Bernard J. Gersh, Ian Hargraves, Nilay D. Shah, Victor M. Montori

**Affiliations:** a Knowledge and Evaluation Research Unit, Mayo Clinic, Rochester, MN, United States; b Robert D. and Patricia E. Kern Center for the Science of Health Care Delivery, Mayo Clinic, Rochester, MN, United States; c Heart Rhythm Section, Cardiovascular Diseases, Mayo Clinic, Rochester, MN, United States; d Division of Health Care Policy and Research, Department of Health Sciences Research, Rochester, MN, United States; e Optum Labs, Cambridge, MA, United States

## Abstract

The data report details the baseline characteristics and observed outcomes among patients included in a large US administrative claims database (Optum Labs Data Warehouse) and those enrolled in the pivotal phase III clinical trials examining apixaban, dabigratan, edoxaban and rivaroxaban versus warfarin for the prevention of cardio embolism (Granger et al., 2011; Cannolly et al., 2009; Patel et al., 2011; Giugliano et al., 2013) [1], [2], [3], [4]. These data are to be interpreted in the context of the linked publication (Noseworthy et al., 2017) [5]. These data illustrate baseline characteristics in patients treated in routine practice and those enrolled in clinical trials. For instance, patients treated with apixaban in practice tended to be slightly older and we more likely to be female than those enrolled in the apixaban clinical trial. Patient treated with rivaroxaban in practice tended to have lower CHADS_2_ scores than those included in the rivaroxaban clinical trial. Overall, and stratified by baseline CHADS_2_ scores, patients treated with NOACs in routine practice had comparable or slightly lower stroke risks than those in the clinical trials. Patients treated with NOACs in routine practice had slightly higher bleeding risk in practice, particularly in high-risk patients with CHADS_2_ ≥ 3, compared to those in the clinical trials. These data may serve as a benchmark for realized outcomes among anticoagulated patients with atrial fibrillation in the United States and may serve as a useful comparison to other datasets or countries.

**Specifications Table**

**Table:** 

Subject area	Cardiology
More specific subject area	Anticoagulation for atrial fibrillation
Type of data	Table
How data was acquired	Administrative claims data
Data format	Analyzed
Experimental factors	OLDW patients with atrial fibrillation receiving treatment with either warfarin, apixaban, dabigatran, or rivaroxaban
Experimental features	Baseline characteristics (age, gender, and CHADs2 score) and event rates (per 100 person years) for ischemic stroke or systemic embolism and major bleeding.
Data source location	US administrative claims, previously published international clinical trial data
Data accessibility	Individual, patient-level from Optum Labs Data Warehouse is not publically available, but summary data are provided in the tables and in the linked publication [5] (DOI: 10.1016/j.ijcard.2017.07.043). All clinical trial data included have been previously published.

**Value of the data**

• These data can serve as a benchmark for ‘real-world’ outcomes in anticoagulated patients with atrial fibrillation.
• These data may be valuable for comparison to outcomes and baseline characteristics observed in other dataset or in other countries.
• These data describe potentially important differences in baseline characteristics between patients treated in routine practice and those enrolled in clinical trials.

## 1 Data

These data are pulled from two sources: (1) a large administrative claims dataset including 107,373 anticoagulated patients with atrial fibrillation, and (2) the pivotal, phase III clinical trials that compared ear of the NOACs to warfarin (Granger et al., 2011; Cannolly et al., 2009; Patel et al., 2011; Giugliano et al., 2013) [1], [2], [3], [4]. Our data report illustrates baseline characteristics (age, gender, and CHADs2 score) as well as event rates for ischemic stroke or systemic embolism and major bleeding, overall and stratified by CHADS2 score.

### 1.1 Experimental design, materials and methods

Using a large U.S. administrative claims database, OptumLabs Data Warehouse (OLDW), which contains privately insured and Medicare Advantage enrollees of all ages and races from all 50 states, we identified adult patients (≥ 18 years) with AF who were new users of oral anticoagulants between July 1st, 2006 and June 30th, 2016, including warfarin, apixaban, dabigatran, or rivaroxaban. Because edoxaban was approved in 2015, there were insufficient sample size to report baseline characteristics and outcomes in edoxaban-treated patients in OLDW. We used the fill dates and days supplied per prescription to determine patients’ treatment episodes. Patients were considered as continuing on treatment as long as they had another fill of the same medication within 90 days of the end of the last treatment episode. We required patients to have at least 12 months of continuous enrollment in health insurance, defined as the baseline, used to capture baseline characteristics. Patients were censored at the end of study period (i.e., 6/30/2016), discontinuation of the medication, switch to another oral anticoagulant, or the end of enrollment in health insurance, whichever occurred first.

The outcomes were inpatient admission for either stroke (effectiveness endpoint, including ischemic stroke and systemic embolism) or major bleeding (safety endpoint). When comparing outcomes to trials, we calculated the event rates per 100 years and stratified patients based on the CHADS2 score (a stroke risk score used in the trials). All the analyses were based on the time to the first event, an approach used in the trials. The study was exempt by the Mayo Clinic Institutional Review Board for approval as we used only pre-existing, de-identified data.
