# Supplementary material for: Baseline characteristics and event rates among anticoagulated patients with atrial fibrillation in practice and pivotal NOAC trials
Source: Data Brief. 2017 Aug 9;14:563–5. doi: 10.1016/j.dib.2017.08.010 (PMC5568872; doi:10.1016/j.dib.2017.08.010)
Supplement: Supplementary file 1 — Supplementary material [file mmc1.docx]

The authors whose names are listed immediately below certify that they have NO affiliations with or involvement in any organization or entity with any financial interest (such as honoraria; educational grants; participation in speakers’ bureaus; membership, employment, consultancies, stock ownership, or other equity interest; and expert testimony or patent-licensing arrangements), or non-financial interest (such as personal or professional relationships, affiliations, knowledge or beliefs) in the subject matter or materials discussed in this manuscript.

Peter A. Noseworthy

Xiaoxi Yao

Bernard J. Gersh

Ian Hargraves

Nilay D. Shah

Victor M. Montori
